# Arbuscular Mycorrhizal Fungi as Core Engineers in Synthetic Microbial Communities: Boosting Plant Growth and Soil Health for Sustainable Agriculture

**DOI:** 10.3390/jof11110769

**Published:** 2025-10-24

**Authors:** Yinan Zeng, Yan Wang, Xueli Wang, Xuemin Jing, Xiangyang Shu, Ping Ren, Weijia Liu, Qinxin Ye, Wei Fu, Zhipeng Hao, Xin Zhang, Baodong Chen, Xia Wang

**Affiliations:** 1 The Key Laboratory of Land Resources Evaluation and Monitoring in Southwest China, College of Geography and Resources, Sichuan Normal University, Chengdu 610066, China; 2 School of Chemistry and Materials Science, Sichuan Normal University, Chengdu 610066, China; 3 State Key Laboratory for Ecological Security of Regions and Cities, Research Center for Eco-Environmental Sciences, Chinese Academy of Sciences, Beijing 100085, China; 4 Heilongjiang Provincial Key Laboratory of Ecological Restoration and Resource Utilization for Cold Region, School of Life Sciences, Heilongjiang University, Harbin 150080, China; 5 Institute of Resources and Environmental Sciences, Chengdu Academy of Agriculture and Forestry Sciences, Chengdu 611130, China; 6 University of Chinese Academy of Sciences, Beijing 100049, China

**Keywords:** SynCom, arbuscular mycorrhizal fungi, AMF microbiome, sustainable agriculture

## Abstract

Bacterial synthetic microbial communities (SynCom) have exhibited significant effects for enhancing plant growth and delivering ecological benefits. However, persistent challenges, including structural instability, limited environmental adaptability, and transient efficacy, remain critical barriers to their practical application. Herein, we propose Arbuscular Mycorrhizal fungi (AMF) as the keystone component to optimize SynCom’s ecological fitness in sustainable agricultural systems. AMF modulate microbiome assembly through hyphal networks, enhancing community stability via facilitative interactions and augmenting nutrient cycling functionalities. This review systematically evaluates methodologies for AMF-based SynCom design and construction, investigates the dynamics of AMF-microbe interactions, delineates plant growth-promoting mechanisms, identifies candidate microbial taxa, and addresses implementation bottlenecks with corresponding strategies. We posit that AMF-Based SynComs represent a transformative management tool for ensuring global food security amid impending climatic perturbations and declining agricultural productivity.

## 1. Introduction

Synthetic microbial community (SynCom) has emerged as a cutting-edge technology derived from plant microbiome research, designed to enhance the effectiveness of current microbial inoculants and address the needs of sustainable agriculture [1,2,3]. SynCom can attain through colonization what a single strain cannot achieve, such as enhanced nutrient uptake and plant growth that surpasses the capabilities of a single strain, even when stacked. Mažylytė et al. (2024) demonstrated that SynComs comprising *Bacillus*, *Pseudomonas*, and *Streptomyces* exhibited emergent properties not observed in individual strains, including synergistic phosphorus solubilization (up to 129.17 mg L^−1^), enhanced secretion of organic acids (such as lactic acid), and the co-regulation of phytohormones like indole-3-acetic acid (IAA). These communal functions contributed to improved wheat growth and phosphorus uptake, thereby reducing the reliance on external fertilizer application [4].

In general, SynCom undergoes two main phases: first, selection and assembly, which involves screening for strains with complementary functions, metabolisms, culturability, and increased antagonism towards each other; second, iterative optimization of ratios, removal of conflicting strains, and addition of missing functions to create a stable and effective simplified community SynCom [5]. In this process, culturability, representativeness, functional complementarity, less antagonism, genetic stability, and environmental and host adaptation of the microbiome serve as the main measurement objectives of SynCom in design and construction [6]. According to these criteria, SynCom, primarily consisting of bacteria, has been extensively studied and has yielded quite favorable results in the field of agriculture. Nevertheless, the mutability and randomness of bacteria hinder the development of bacterial SynCom.

Fungi achieve a better SynCom by exploring soil nutrients more efficiently through their mycelial networks, offering greater stability and adaptability to various soil conditions, including drought, salinity, and heavy metals, compared to bacteria. This makes fungi more suitable as a core microbial component in SynCom strains [7,8,9]. Core microbial strains are key in forming plant communities. They recruit native microorganisms with diverse functions and prevent pathogenic bacteria and pests, benefiting the host plant [10,11].

Arbuscular mycorrhizal fungi (AMF) are soil symbionts that could colonize the roots of more than 70% of terrestrial plants [12]. In arbuscular mycorrhizal symbiosis, AMF and plant roots exchange low-P exudates (<500 Da) and fungal Myc factors (<3.5 kDa) to establish colonization. AMF secretion controls plant recognition of its induced symbiotic receptor-like protein kinase, SYMRK-DMI1-DMI3, which cascades to regulate the formation of clumped mycelia to facilitate nutrient exchange with plants. The colonized plant cells express MtPT4, a phosphate transport protein, to stabilize the nutrient exchange relationship with AMF. At the same time, neighboring cells release SCP1, Small Cysteine-Rich Secreted Protein, to inhibit local immune responses and GST, Glutathione S-Transferase, to clear metabolic toxins and ensure that the symbiosis proceeds stably. Systemically, lateral root growth increases, and phosphate-starvation responses decrease. These signals, found in 400-million-year-old fossils and over 80% of vascular plants, support plant growth and make AM symbiosis the most ancient terrestrial mutualistic relationship [13]. The symbiosis benefits plants by speeding soil organic matter mineralization, increasing stress tolerance, and shaping the microbiome to enhance stability and longevity [14,15]. The AMF hyphosphere, affected by hyphal exudates, has unique physical and chemical properties [16,17], making its microbiome distinct from the rhizosphere microbiome influenced by plant root exudates.

Considering the potent functionalities of AMF, the commercial market size of AMF bioinoculants has approached US$995 million [18]. Nevertheless, the widely available AMF bacterial inoculants on the market typically exhibit disadvantages such as low activity, limited colonization capacity, and subpar results. In a review by Koizol et al. of 302 commercial AMF mycorrhizal inoculants worldwide, the average colonization rate was less than 9%, markedly lower than that of laboratory-cultured inoculants. Furthermore, only 12% of these commercial inoculants displayed adequate mycorrhizal adaptation to promote plant growth effectively [18]. Given the insufficient stabilization of bacterial SynCom and the ineffectiveness of commercial AMF bacteriophages, it becomes necessary to combine AMF with SynCom. This approach involves integrating bacteria, such as plant growth-promoting rhizobacteria (PGPR), or other AMF species to form a more effective AMF-based SynCom [19]. This innovative biofertilizer aims to enhance agricultural yield and sustainability.

This study focuses on the AMF-based SynCom: (1) Designed and construction methods of AMF-based SynCom. It covers common forms of AMF inoculants and large-scale production methods. (2) Expounded AMF interaction with fungi or bacteria. (3) Revealed AMF-based SynCom promotion and mechanism on plants. (4) Discussed the theoretical basis and application method of AMF SynCom, as well as the limitations of the development and application of AMF SynCom at the present stage. This review provides a systematic framework for designing and constructing AMF-based SynCom, advancing its potential applications in agriculture and offering valuable insights for future research and large-scale production.

## 2. Design and Preparation of AMF-Based SynCom

The application of AMF-based SynCom is presently confined to laboratory settings and has not yet attained comprehensive agricultural deployment or undergone extensive field trials to evaluate its effects [20]. Based on experimental outcomes with AMF-based SynCom and subsequent analyses, this work proposes methodologies for their design and development, as well as production techniques for various AMF types and their unique characteristics.

### 2.1. Taxonomic Basis of AMF to Engage in SynCom

To accurately regulate AMF-based SynCom, it is essential to comprehend the classification of AMF and its core species, which play a dominant role in promoting plant growth. Furthermore, this understanding serves as the foundation for investigating the interactions between AMFs and other microorganisms, thereby facilitating a comprehensive understanding of the mechanisms underlying AMF-based SynCom.

Currently, the AMF group is under the phylum *Glomeromycota* and the subgroup *Mucoromyceta*. According to the 2024 AMF phylogeny at http://www.amf-phylogeny.com/ (accessed on 20 May 2024), at least 352 species have been recorded to date [21] (Table 1). Although AMF classification is evolving, designing AMF-based SynCom and understanding the species’ characteristics and performance under varying conditions can help select optimal species combinations for crop development and agricultural efficiency.

Of the 352 AMFs identified, several researchers uncovered the varying ability among AMF species to enhance growth or defense responses to plants [22,23]. Most AMF are in the subphylum Glomeromycotina. Some experts believe that species from the family Glomeraceae are more effective in protecting plants against diseases, whereas those from Gigasporaceae are more proficient in promoting plant growth [24,25]. Furthermore, the genera *Rhizophagus*, *Funneliformis*, and *Glomus* are extensively utilized in agriculture due to their broad host range and adaptability to various soil conditions [26], demonstrating their potential for selection in AMF-based SynCom.

Species within the genus *Rhizophagus*, such as *Rhizophagus irregularis*, exhibit a high colonization rate and are capable of forming an extensive hyphal network. These characteristics facilitate their effective mediation of nutrient acquisition from the surrounding soil volume around the root system [27]. Species in the genus *Funneliformis*, exemplified by *Funneliformis mosseae*, demonstrate a diversity in host colonization and exhibit resistance to various abiotic and biotic stresses in the environment [28]. Members of the genus *Glomus* are widely acknowledged for their potential to enhance plant drought tolerance and promote growth under conditions of nutrient deficiency [29]. The aforementioned AMF strains, with notable plant growth-promoting and stress-mitigating functions, constitute a valuable microbial resource pool for the development of AMF-based SynCom.

As the core constituent strains of AMF-based SynCom, some incompatibilities among AMFs need to be taken care of so as not to cause damage to the stability and effectiveness of AMF-based SynCom. In the experiment conducted by Malicka et al. (2021), *Claroideoglomus walkeri*, *Diversispora varaderana*, and *Funneliformis caledonium* were used to inoculate *Lolium perenne* under phenol and PAH stress. The results showed that, when co-inoculated as a mixed inoculum, *C. walkeri* rapidly dominated the root niche, leading to excessive sporulation and carbon sequestration. This competitive overgrowth suppressed the establishment and function of the other two AMF species, ultimately resulting in significantly reduced plant height, biomass, and root weight compared to single-species inoculations. The mechanism is likely linked to *C. walkeri*’s rapid intraradical mycelium (IRM) expansion and high spore production, which disproportionately consumed host-derived carbon, out-competed *F. caledonium* and *D. varaderana* for root space and nutrients, and disrupted the carbon–phosphorus exchange balance. This imbalance shifted resource allocation from plant growth to AMF reproduction, thereby undermining the functional complementarity expected from the mixed inoculum and inducing an ecological imbalance between the AMF partners [30].

**Table 1:** Current and evolving taxonomic classification of arbuscular mycorrhizal fungi [21,29,31].

Class	Order	Family	Genera
Archaeosporomycetes	Archaeosporales	Ambisporaceae	*Ambispora*
		Archaeosporaceae	*Archaeospora*
		Geosiphonaceae	*Geosiphon*
		Polonosporaceae	*Polonospora*
Glomeromycetes	Diversisporales	Acaulosporaceae	*Acaulospora*
		Diversisporaceae	*Corymbiglomus*, *Desertispora*, *Diversispora*, *Otospora*, *Redeckera*, *Sieverdingia* and *Tricispora*
		Gigasporaceae	*Bulbospora*, *Cetraspora*, *Dentiscutata*, *Fuscutata*, *Gigaspora*, *Intraornatospora*, *Paradentiscutata*, *Racocetra* and *Scutellospora*
		Pacisporaceae	*Pacispora*
		Sacculosporaceae	*Sacculospora*
	Entrophosporales	Entrophosporaceae	*Entrophospora*, *Albahupha* and *Alborhynchus*
	Glomerales	Glomeraceae	*Complexispora*, *Dominikia*, *Epigeocarpum*, *Funneliformis*, *Funneliglomus*, *Glomus*, *Halonatospora*, *Kamienskia*, *Silvaspora*, *Microdominikia*, *Microkamienskia*, *Oehlia*, *Nanoglomus*, *Orientoglomus*, *Rhizoglomus*, *Rhizophagus*, *Sclerocarpum*, *Sclerocystis* and *Septoglomus*
Paraglomeromycetes	Paraglomerales	Paraglomerales	*Innospora* and *Paraglomus*
		Pervetustaceae	*Pervetustus*

### 2.2. Design and Construction of AMF-Based SynCom and Large-Scale Production Approaches for AMF-Based SynCom

#### 2.2.1. Design and Construction of AMF-Based SynCom

The design and construction of AMF-based SynCom followed general SynCom principles: (1) screening strains and forming a microbiome based on functional and antagonistic relationships; (2) observing interactions through antagonistic experiments; and (3) verifying effectiveness via potting or field tests. The goals are: (1) higher yield than single inoculation; (2) improved stability of inter-root microbes; (3) richer ecological diversity; and (4) wider application [16]. Schmitz developed a five-strain SynCom through screening 15 desert bacteria for salt tolerance in tomatoes, evaluating various combinations under 200 mM NaCl. This resulted in a 34% increase in biomass, improved ion balance, higher yield compared to individual strains, and stable growth in non-sterile soil [31]. A reliable methodology, such as ‘top-down’ or ‘bottom-up,’ is essential for optimizing microbiome design [32].

The “top-down” methodologies employ the “cry for rescue” mechanism of the host plant, which is placed within a deliberately designed stressful environment to iteratively recruit and mold microbial communities. The research concentrates on steering the evolution of the existing microbial community associated with the host plant in a specific direction by applying biotic or abiotic stresses encountered in the environment. Throughout the evolutionary process, core microbial strains capable of coping with or managing the corresponding stresses will gradually dominate the community, leading to the formation of specialized functional microbiomes. Subsequently, the dominant core microorganisms under stress are isolated and characterized, and differences in microbial abundance between phenotypic samples are compared to mitigate the influence of chance events. This process simplifies the indigenous microbial community into the desired SynCom through selective screening [17,33]. Because it effectively represents a scaled-down version of the native soil microbiota, SynCom constructed via the “top-down” approach can be more seamlessly integrated with the native microbiome following field application. This method enhances the proliferation of functional microorganisms within the indigenous microbiome, enabling them to evolve into the dominant strains, which surpasses inoculation with unfamiliar microorganisms alone. The “top-down” approach is particularly advantageous in scenarios where knowledge regarding the assembly and function of microbial communities is limited or when a functional SynCom must be rapidly developed. It facilitates the swift amelioration of stresses and challenges faced by crops, thereby resulting in tangible increases in crop yield and environmental benefits. Nonetheless, its lack of consideration for microbial interactions, reliance on existing microbial communities, and absence of functional validation may compromise the functionality and long-term stability of the constructed SynCom at somewhat uncontrollable [33].

The “down-top” or “reductionism” approach involves the deliberate selection and assembly of specific functional strains to construct the SynCom directly. The core to this methodology resides in the precise selection of significant functional organisms and the appropriate assembly ratios, which directly impact the efficacy of the final SynCom. Technologies such as macrogenomics, transcriptomics, metabolomics, and others are frequently employed to detect and screen functional genes of the strains (such as putative sugar transporter gene (*fruT* and *gluT*) and phosphatase genes (*acp1* and *alp*) detected from *Rhizophagus irregularis*), while additionally exploring metabolic mechanisms through existing literature, to obtain powerful strains with complementary metabolisms [2,3,34]. In the development of the “down-top” method, antagonism experiments are also conducted on the selected strains to evaluate interspecies relationships and the ratios of strains within the consortium. It is important to recognize that during this process, these strains are not derived from an already integrated natural community and may exhibit unforeseen antagonistic effects [3]. The “down-top” approach is well-suited to scenarios that demand meticulous control of the microbiome and its function. Moreover, the establishment of the fundamental microbiome-building process and modeling facilitates the subsequent construction of the microbiome structure, thereby enabling the addition and replacement of novel strains to address diverse challenges [35]. The “down-top” approach is highly suitable for applications in industrial manufacturing, where meticulous regulation of bacterial microbiome and their operations is imperative. Furthermore, the development of fundamental microbiome-building processes and modeling supports the subsequent construction of microbiome structures, thereby facilitating the integration and replacement of novel strains to address various challenges. However, to select and assemble microorganisms with efficiency and precision, considerable demands are imposed on multi-omics analysis, microbial culturing, and the expertise of operators. These requirements have impeded its broader adoption [36].

Guided by two methodologies, a SynCom design method based on metabolic complementarity has been proposed and sees great potential in practice [2]. The approach involves utilizing metabolic complementarity as a screening criterion, with the Biosynthetic Support Score (BBS) and microbial nutrient support capacity to the host serving as the primary indices. These metrics are employed to screen and streamline a functionally complete, redundancy-ranked, and synergistic minimal community from the complex system microbiome [37]. During the construction process, it is essential to gather the macro-genome assembly genomes (MAGs), followed by conducting metabolic modeling through the reconstruction of each MAG’s Genome-Scale Metabolic Network (GSMN) [38]. Subsequently, key compounds necessary for “plant-microbe” interactions should be identified, including essential metabolites such as amino acids, nucleotides, and cofactors, as well as plant-interacting compounds like phytohormones and organic acids [39]. On one side, identifying the minimal set of species capable of producing all target compounds to summarize all metabolic functions via a minimum community screening using m2m mincom [40]. Conversely, we can identify strains exhibiting complete plant growth-promoting traits (PGPTs) based on key genes, such as the nitrogen-fixing gene *niFH*, the potassium-solubilizing gene *kdpA-E*, and the IAA metabolizing pathway gene *trp*, among others (for example, Nitrogen-fixing bacteria *Metallosakonia intestini* [41]. Finally, through metabolic complementarity analysis, we could compute the competition and complementarity indices between species to select highly complementary pairs [42]. Additionally, through plant-microbe mutualism analysis, we can determine the biosupport score and metabolic complementarity index to identify species with strong mutualistic relationships with plants. Designed by the Metabolic Complementarity Approach, SynCom offers a standardized operational framework and a computational template for subsequent production and adaptation, owing to its straightforward structure following precise measurements and microbiome screening during development. Moreover, it presents significant potential for applications such as the development of SynCom inoculants, crop-specific microbiome design, and microbial resource exploration in extreme environments.

The effectiveness of the SynCom’s formulation and assembly could be confirmed through both potting and field experiments. These experiments evaluate various plant growth parameters, including plant height, fresh weight, and dry weight. In addition, the experiments included the analysis of soil and root-associated microbial communities. Other factors examined included nutrient levels of nitrogen, phosphorus, and potassium, enzyme activity, and the structure of the microbial community, to accurately evaluate the impact of the SynCom [2,43,44].

#### 2.2.2. AMF-Based SynCom Agent and Production Methods

**Figure 1:**
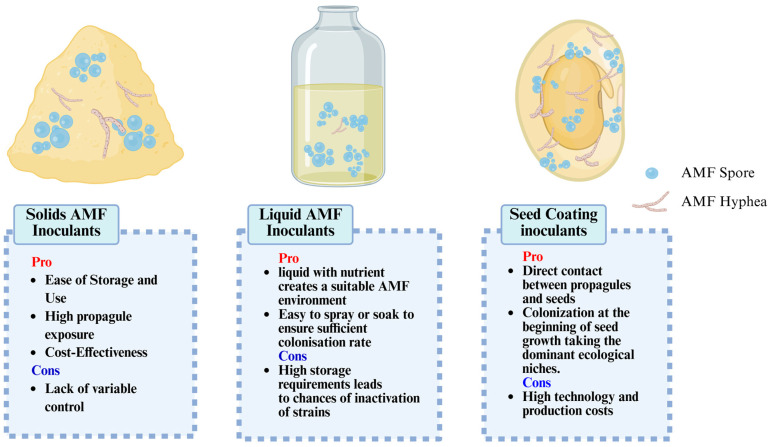
Solids, Liquid, Seed-Coating AMF inoculants and their features [45,46,47,48].

Amendments of AMF can be classified into solid, liquid, seed-coated, and spore capsule categories based on the carrier material (Figure 1). Solid AMF amendments are preferred in AMF-based SynCom applications due to their benefits of easy storage, high strain activity, cost efficiency, and simple application. As a result, solid AMF amendments make up about 60% of inocula, while liquid amendments represent around 29% [45].

**Figure 2:**
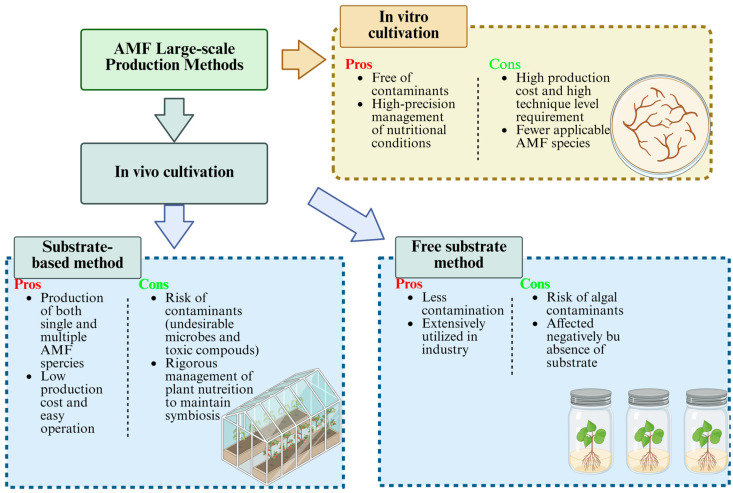
AMF Large-scale Production Methods and Their Pros and Cons [49,50,51,52].

To achieve large-scale and cost-effective AMF propagation, the methods of large-scale production are categorized into in vivo and in vitro. The in vivo method includes substrate and substrate-free approaches [36,48] (Figure 2). Among them, the substrate method is the most widely used in production for its low cost and ease of handling.

#### 2.2.3. Co-Culture Strategy: Essential for Synchronized Amplification of AMF and Companion Bacteria

In the context of AMF-based SynCom production, following the selection and compatibility testing of strains, it is crucial to ensure the integrity of the entire microbial community. Conventional SynCom production entails the straightforward mixing of microorganisms after individual cultivation, thereby facilitating control over the structure and function of the microbial assemblage. Nevertheless, this method presents limitations related to the ratio of strains within the community and the development of synergistic relationships [53,54]. Its inherent limitation in regulating strain proportions and synergistic relationships inevitably leads to prolonged intra-community regulation during system integration. The integrity of the microbial community may be compromised during the complex process of system integration, thereby impairing its functionality and adaptability [55]. Research suggests that microbial responses to environmental modifications are not immediate, with a lag of up to three years, and that the composition of the microbial community exerts a more significant influence on functional efficiency than microbial abundance at the time of community establishment [56].

The co-culture method is regarded as a more efficacious approach for generating AMF-based SynCom, as it endeavors to maintain the integrity of the SynCom. This methodology integrates traditional soil culture of AMF with other strains within a meticulously designed SynCom. It entails the sterile inoculation of the host plant in a controlled environment, resulting in the collection of solid pellets or bacterial agents that encompass the entire AMF-based SynCom. This strategy leverages AMF and its inherent community regulation mechanisms, permitting the community to develop and self-regulate naturally during cultivation. Strains are pre-screened for compatibility, significantly reducing antagonism and arbitrary functional interactions, thereby ensuring the stability and reliability of the co-culture approach [57].

Furthermore, the co-culture method offers notable advantages for the production and quality of AMF-based SynCom. Incorporating additional target microorganisms into AMF breeding strains enhances compatibility with various SynCom configurations, including AMF-AMF combinations, AMF paired with synergistic bacteria, and other potential assemblies [55]. This approach also prevents community non-integration by simply mixing the bacterial microbiome, thereby allowing community integration to occur naturally during cultivation [57,58]. In instances where AMF-based SynCom predominantly consist of bacteria and AMF, which require bacterial cultivation, the co-culture propagation method can leverage the soil system’s complexity and the regulatory mechanisms of AMF. Its primary objective is to mitigate uncertainties related to community function that may arise from multi-generational propagation and the consequent loss of functional genes due to mutation. Additionally, this method aids in maintaining community diversity, which is often endangered by antagonism among target bacteria, thus preserving community integrity [57].

Furthermore, for producers, the community shaping selection of complex soil systems and AMF significantly reduces the laborious steps involved in adjusting the strain ratio during the assembly of traditional isolation and culture methods, thereby streamlining the design and manufacturing process. Nonetheless, the co-culture approach encounters challenges related to scalability and cultivation efficiency: AMF must grow symbiotically with plants, which requires considerably more time than bacterial preparations that can be harvested within a few days, leading to operational complexities, elevated costs, and increased risks. Moving forward, selecting host plants that can be rapidly harvested and developing cost-effective, intensive culture techniques for AMF are anticipated to serve as viable solutions.

## 3. AMF-Based SynCom Interaction and Plant Growth Promotion

**Figure 3:**
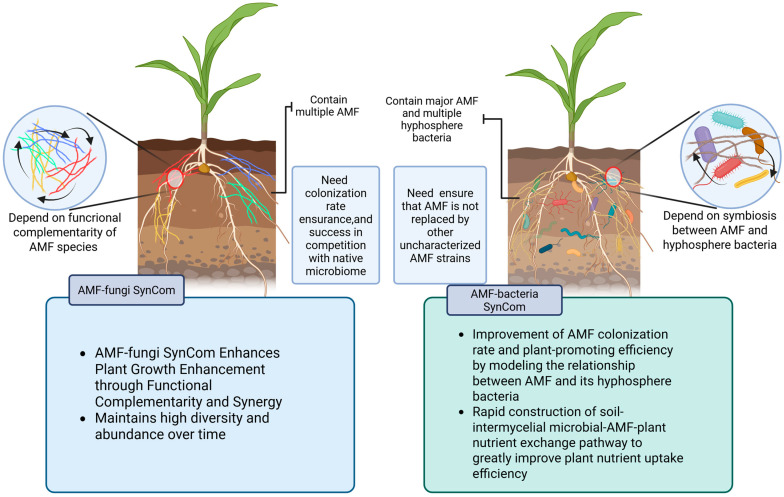
Comparison of AMF-based SynCom mainly constructed by fungi or bacteria and AMF applications [59,60].

Adopting multiple AMFs with multiple hyphosphere or rhizosphere core strains for SynCom construction in future studies is a promising way to enhance the effectiveness and sustainability of AMF-based SynCom (Figure 3). The bio-interaction among AMF and other microorganisms, including fungi and bacteria, is fundamental. Clarifying the relationships between AMF and other microorganisms within the hyphosphere and rhizosphere establishes a comprehensive theoretical foundation for the construction and application of AMF-based SynCom. Furthermore, it offers essential guidance for understanding, analyzing, and the precise development of SynComs (Table 2).

### 3.1. Interactions in AMF-Based SynCom Constructed by Fungi and Its Plant Growth Promotion Effects

The diversity of AMFs provides rich microbial resources for the construction of AMF-based SynCom mainly constructed by fungi (Table 1), which enables the selection of strains from different AMFs and the construction of stable and efficient SynCom. For the simpler only AMF-Mycorrhizal Fungi SynCom of functional complementarity and synergy between AMF species play a major role. Jansa et al. compared the effects of simultaneous colonization by different AMF with single AMF colonization on plant nutrient uptake and growth. In mixed inoculation of both AMFs, *Glomus intraradices* and *Glomus claroideum* [59]. It was found that only when two AMFs with functional complementarity, such as *G. intraradices* and *G. claroideum*, were inoculated, did their AMF communities retain high diversity and richness. *G. intraradices* is mainly able to take up phosphorus from soils further away from the root system, whereas *G. claroideum* takes up phosphorus mainly from the vicinity of the root system [62]. This difference in function and ecological niche allows for stable function and good diversity and richness over time [16]. In groups without functional complementarity, the richness and functionality of the microbial community decreased to varying degrees. While this loss is a normal part of community succession, it still negatively impacts the community’s function and adaptability.

To the co-operation among AMF species, the interactions between *Rhizophagus* and *Funneliformis* may be competing for the different colonization sites in the root system, which present as *Rhizophagus* tends to colonize the apical region, while *Funneliformis* is more colonized in the root hair region. This colonization pattern reduces direct competition for resources among AMFs [63]. And evidence supports that such AMF species can interact synergistically, especially if their nutrient acquisition strategies are complementary [64]. Thonar et al. [65] reported the ability of *Rhizophagus. irregularis* and *Funneliformis. mosseae*, when co-inoculated, to enhance phosphorus uptake in Medicago truncatula plants compared to their single inoculations. A synergistic effect that has been related to functional complementarity of the two AMF species tested, which would benefit differentially from soil phosphorus pools, increasing plant P acquisition efficiency [63]. Researchers reported that co-inoculation increased plant biomass by 20% and phosphorus content by 30% over the non-inoculated control, thus pointing out the potential of AMF species to complement each other in facilitating nutrient acquisition.

The activation of system resistance and the enhanced resilience to biotic and abiotic stresses, resulting from the cooperation of AMF species, must not be overlooked. Research conducted by Liu et al. [66] and Parvin et al. [67] involved the application of AMF to peanut (*Arachis hypogaea* L.) and rice (*Oryza sativa* L.) seedlings under salinity stress. Following a growth period of seven to eight weeks under controlled conditions, plants inoculated with a SynCom comprising *Rhizophagus irregularis* SA, *Rhizophagus clarus* BEG142, *Glomus lamellosum* ON393, and *Funneliformis mosseae* BEG95, or with *Acaulospora laevis* BEG13 and *Gigaspora margarita* BEG34, demonstrated significantly higher leaf relative water content, photosynthetic rate (Pn), and K^+^/Na^+^ ratio. Additionally, these plants exhibited reduced levels of malondialdehyde (MDA) and reactive oxygen species (ROS) compared to non-inoculated controls. Such physiological enhancements were correlated with elevated activity of antioxidant enzymes (SOD, CAT, APX, G-POD) and increased accumulation of osmolytes, including soluble sugars and free amino acids, thereby suggesting that AMF promotes systemic redox homeostasis and ionic regulation, which in turn enhances the plants’ tolerance to salinity.

The competitive dynamics among AMF species are of significant importance. Particularly under conditions of nutrient deficiency or other environmental stresses, competing species may exclude each other from specific niches. Less competitive species tend to be excluded, especially noting that phylogenetically close species possess similar requirements within fundamental niches, resulting in competition within the ecological context niches [68,69]. When competing relationships between different AMF species had been tested in a field setup, Bonfante et al. found that a single dominant species of AMF was capable of reducing other species’ colonization potentials by up to 50%, thus altering the AMF community composition, thereby affecting the ecosystem services that these fungi may provide [70]. Interactions among plants and diverse AMF species are inherently complex, involving numerous mechanisms of both chemical and biological nature [20]. For instance, AMF species can release chemical signals that either attract or repel other AMF species, which is a phenomenon influenced by soil pH, nutrient availability provided by the plant, and the presence of specific bacterial taxa [71]. In addition, the competitive outcome may also depend on the physical architecture of the mycelium: species forming pervasive networks may competitively outperform species developing poorly interconnected networks [72,73].

Complex dynamics among AMF species involve not only competition and cooperation but also a complex interplay of different ecological strategies, which largely affect nutrient cycling in the soil and plant health. The identity of the co-colonizing AMF and their inoculum density determine if they compete or promote each other during root colonization of the host plant [74], which significantly affects the effectiveness of the community in promoting plant growth.

#### Trichoderma

*Trichoderma* (Ascomycota, Hypocreaceae) encompasses over 500 officially recognized species distributed globally in soils, the rhizosphere, and decaying organic matter [75]. It exhibits a pronounced synergistic interaction with AMF. Moreover, it constitutes the non-AMF present within the soil ecosystem, which is crucial when formulating AMF-based SynCom. These SynComs frequently exploit the dual function of *Trichoderma* as both a mycoparasite and a plant-growth-promoting fungus (PGPF), the latter of which solubilizes phosphorus, synthesizes phytohormones such as auxins, gibberellins, and cytokinins, and induces systemic resistance (ISR) via jasmonic acid and ethylene signaling pathways [76].

The study also investigates the relationship between *Trichoderma* and AMF. It not only significantly enhances AMF colonization but also demonstrates a notable antimicrobial effect against numerous pathogenic bacteria [77,78]. Direct antagonism is mediated through the secretion of cell wall-degrading enzymes (such as chitinases and glucanases) and secondary metabolites—including 6-pentyl-α-pyrone, gliotoxins, and peptaibols—that inhibit pathogens such as *Fusarium* spp., *Rhizoctonia solani*, and *Sclerotium rolfsii* [76,79]. Indirect antagonism involves competition for nutrients and space, as well as the activation of plant defense mechanisms.Al-Asbahi et al.l. observed that the mRNA level of AM protein was 2.5 times higher than the control in wheat leaves treated with *Trichoderma*. They hypothesize that *Trichoderma* indirectly boosts AM protein mRNA expression by releasing volatile signaling molecules that enhance AMF colonization and plant nutrient uptake. Al-Asbahi et al.l. conducted experiments showing the effects and mechanisms of AMF and *Trichoderma* on melon wilt [78]. *Trichoderma* inoculation notably increased the colonization of *G. intraradices*, *G. constrictum*, and *G. claroideum*. Additionally, both *Trichoderma* and AMF induced systemic resistance in plants, antagonizing the pathogen and reducing the infection rate in co-inoculated plants to below 5% [80].

In practice, when non-AMF are present and exhibit synergistic effects with AMF, there is an increase in the abundance of AMF within the inter-root environment, thereby establishing it as the dominant species within the community. Future SynCom engineering may exploit strain-specific traits; for example, the gamma-irradiated mutant *T. harzianum* TM enhanced barley drought tolerance by more effectively elevating root auxin levels and respiratory enzyme activities compared to the wild-type strain [76]. Alternatively, we can follow research on *Trichoderma* by selecting fungal strains that are synergistic with AMF to create a robust SynCom, mainly constructed by fungi that can counteract the negative effects on AMF in the soil environment. By exploring the principle of AMF interactions with other fungi, such as other AMF species or *Trichoderma*, we can identify suitable and powerful microorganisms to add to AMF-based SynCom, thereby constructing a more powerful SynCom that provides directional guidance.

### 3.2. Interactions Between AMF and Bacteria and Their Effects on Promoting Plant Growth

The AMF-based SynCom composed of bacteria and AMF mimic the natural interaction in which AMF shapes the hyphosphere and rhizosphere bacterial community and their symbiotic relationships. In this construct, AMF serves as the backbone, with core hyphosphere and rhizosphere bacteria selected for their strong synergistic effects with the selected AMF strains. The goal is to artificially create a simplified microbiome that enhances AMF colonization rates and plant growth performance by pre-establishing a symbiotic relationship between AMF and hyphosphere and rhizosphere bacteria [81,82].

*Streptomyces*, a prevalent and dominant bacterial genus in the AMF mycelial interstitium, is intimately associated with the metabolism and function of AMF. The diverse soil bacteria play a critical role in underpinning the synergy effect with AMF. AMF recruit and feed bacteria by releasing carbon sources, which in turn support AMF through various functions [83]. This mutualistic symbiosis not only extends the plant-AMF continuum but also forms a complete material exchange network involving plants, AMF, and bacteria. This network significantly increases the efficiency and stability of material and signal transfer within the ecosystem [84]. A deeper understanding of the mechanisms underlying AMF-bacteria interactions will facilitate the systematic and precise construction of effective AMF-based SynCom, mainly constructed by bacteria and AMF.

Further reinforcing this point, the mutualism between AMF and bacteria benefits plant nutrition. Co-inoculating AMF with bacteria like *Streptomyces* and *Pseudomonas* increases plant P and K uptake by 69.86% and 25.56%, respectively, compared to AMF alone [85]. This enhancement is due to the dual action of bacterial mineralization, such as *Streptomyces*, which converts up to 83 ± 7% of phytic acid into bioavailable P—and AMF-mediated polyphosphate chain transport [86]. In addition, *Rhizobium* co-inoculation enhances the mycelial network by 93.7%, establishing a bidirectional exchange loop in which plants allocate 18–30% of photosynthates (e.g., alginate, fructose) to the AMF-based SynCom, mainly constructed by bacteria and AMF, which reciprocates by channeling mineralized nutrients via PT transporters (e.g., Pho84, VTC2/4) [87].

AMF and PGPR collaboration enhances plant stress responses and resilience [88]. Yang et al. studied soybeans co-inoculated with AMF (*Rhizophagus intraradices*) and PGPR (*Pseudomonas psychrotolerans*) under acidic soil stress (pH 4.54, low phosphorus, high aluminum). After 59–104 days, the plants grew 22% taller, with an increase in nitrogen-fixing bacteria, *Streptomyces*, and enhanced synthesis of unsaturated fatty acids. The AMF network enhanced root uptake, recruited aluminum-tolerant PGPR and nitrogen-fixing bacteria, and improved nitrogen and phosphorus utilization. It also increased unsaturated fatty acids and steroids, which improved membrane stability and antioxidants. The fungus *Nigrospora oryzae* stimulates antimicrobial groups like Streptomyces, creating a defense system that reduces aluminum toxicity and promotes soybean growth in acidic soil conditions [89].

**Figure 4:**
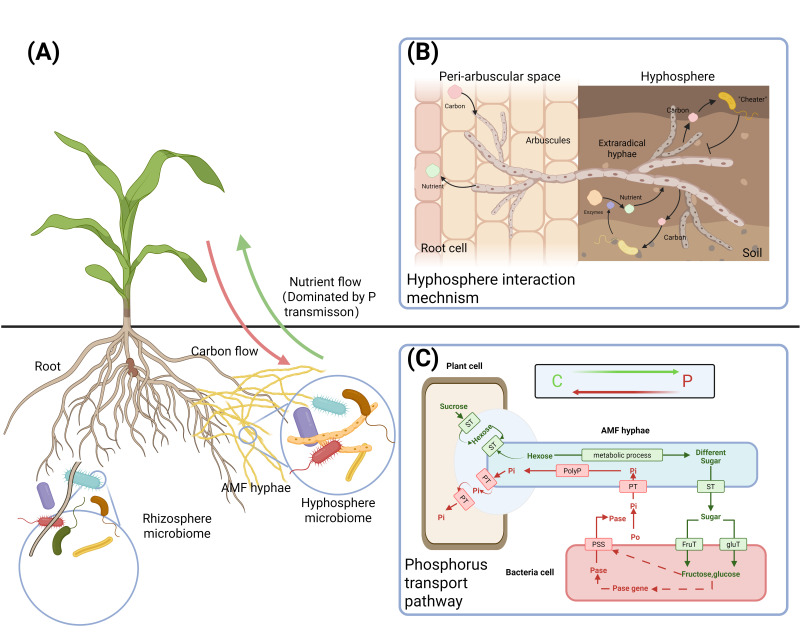
The substance transport relationship between AMF and the continuum formed by the collaborating bacteria [94]. (**A**) shows the spatial relationship between the various parts of the continuum and the overall direction of material transport in the continuum (carbon and mineral nutrient flow); (**B**) shows the mechanism of bacterial-AMF interactions in the mycelium, including direct and indirect regulation; (**C**) shows in detail the mineralization transport pathway of P, which accounts for most of the nutrient transport, in the plant-AMF-bacteria continuum.

AMF interacts with bacteria primarily in the hyphosphere [90]. There are two different mechanisms of cooperation between AMF and bacteria, known as direct and indirect reciprocity, resulting from the exchange of metabolites. (Figure 4B). Direct reciprocity refers to a direct nutrient exchange between AMF and bacteria, where AMF release exudates in the hyphosphere to feed a specific microbiome. It could bring nutrients to AMF directly to strengthen the fitness of AMF symbiosis. But some “cheaters” bacteria, who benefits from AMF without making a personal contribution, pose a risk for direct reciprocity. The indirect reciprocity allows bacteria to dominate the cooperation, which could interfere with or suppress those “cheater” bacteria gaining access to nutrients, harming the mutually beneficial relationship. Through those two mechanisms, Reciprocal relationships between AMF and the hyphosphere microbiome are maintained, and they work consistently and efficiently [60].

AMF and bacteria within SynCom establish a continuous substance exchange consortium with plants, supplying mineral nutrients in exchange for carbohydrates to sustain physiological activity. In this nutrient exchange process, phosphorus predominantly governs the trophic transfer of mineral elements. The transport of phosphorus is mediated by the mycelial network, serving as a conduit for phosphorus movement into the plant. This occurs through the synthesis of polyphosphate chains within the extraradical mycorrhizal network and their subsequent translocation into the intraradical mycorrhizal network. Subsequently, the hydrolysis of polyphosphate within the arbuscules releases phosphate ions into the hyphosphere, where they are absorbed by plant phosphate uptake mechanisms transporters [86].

The rhizosphere microbiome comprises bacteria residing in the rhizosphere, primarily under direct regulation by rhizosphere secretions. (Figure 4). The rhizosphere microbiome plays an important role in plant nutrient uptake and other aspects, like hyphosphere bacteria. AMF can affect the composition and activity of the rhizosphere microbiome by regulating the levels of hormones in plants, such as growth hormone and gibberellin, and plant gene expression, which affect plant growth and development. This regulation results in altering the plant’s root secretions [91,92]. In Du et al.’s study, they found that AMF up-regulated gene expression associated with starch and sucrose metabolism, phenolic metabolism, terpene biosynthesis, fatty acid synthesis, and hormone metabolism under low phosphorus conditions, which are remarkably higher than high phosphorus conditions [93].

Among the many microorganisms in the hyphosphere and rhizosphere, some core strains with universal strong synergistic effects with AMF are the cornerstone of SynCom construction. *Streptomyces*, *Pseudomonas*, and *Bacillus* spp. have been studied extensively in the past and are often co-studied with AMF. They share unique collaborative and mutualistic mechanisms with AMF, which include the promotion of carbon-phosphorus nutrient cycling in AMF, the co-enhancement of plant disease resistance and soil quality, the enhancement of AMF colonization, and the promotion of shaping of hyphosphere microbiome. We recommend prioritizing these strains when designing and constructing AMF-based SynCom.

#### 3.2.1. Streptomyces

*Streptomyces*, a prevalent and dominant bacterial genus within the AMF mycelial interstitium, is closely associated with the metabolism and function of AMF. Jin et al. observed that *Streptomyces* comprised an average of 77.5% of the interstitial bacterial community in the AMF mycelial interstitium. Conversely, the proportion of *Streptomyces* in the control group, which did not receive AMF supplementation, was only 24.3%. This phenomenon was attributed to *Streptomyces*’s preferential utilization of four carbon sources supplied by AMF, including glucose, fructose, and alginate. Furthermore, *Streptomyces* demonstrated a strong affinity for alginate, a sugar that is less commonly utilized by bacteria as a carbon source [95]. In addition, the expression of several related transport, metabolism-related genes (*ThuE*, *ThuF*, *ThuG*, *OtsB*, *TREH*, etc.) was significantly up-regulated in the presence of AMF [96].

Concurrently, *Streptomyces* demonstrated significantly enhanced mineralization and transport capabilities for organic phosphorus and phosphorus, exhibiting levels 37 times greater than those observed in other strains [94]. It revealed the ability of *Streptomyces* to convert various forms of organic phosphorus (e.g., phytic acid) into inorganic phosphorus and to establish a phosphorus transport pathway in collaboration with AMF (Figure 4C), thereby markedly increasing phosphorus acquisition of plants within soil environments. This phenomenon can be attributed to the *Streptomyces* genome’s possession of a higher number of genes associated with phosphorus metabolism compared to other strains. These include *phoD* (dominant organic phosphorus mineralization), *gcd* and *gdh* (inorganic phosphorus lysis genes), and phosphorus transporter genes such as *pstS*, *pstC*, and *pstB* [97]. It has been demonstrated that *Streptomyces* displays elevated expression levels of the aforementioned genes in the context of coexistence with AMF, resulting in the rise of the synthesis of substances such as the phosphorus transporter protein Pho84 and the polyphosphate transporter protein VTC2/4 [98].

The role of *Streptomyces* in phosphorus cycling not only benefits plant growth but also helps stabilize soil ecosystems. Their secretion of melanin and polysaccharides, along with AMF glomalin, led to a 13.1% increase in >2 mm soil aggregates, which improves water retention as shown by an 18% rise in porosity [99]. Additionally, *Streptomyces necromass* accounted for 22–35% of stable soil organic matter (SOM), while the turnover of AMF hyphae released labile carbon pools that sped up SOM decomposition by 29% [91]. This dual action balances carbon sequestration and nutrient availability, which is critical for sustainable soil health.

Additionally, *Streptomyces necromass* accounted for 22–35% of stable soil organic matter (SOM), while the turnover of AMF hyphae released labile carbon pools that sped up SOM decomposition by 29% (Figure 4B). This is evidenced by the secretion of antimicrobial substances such as albaflavenone [100], which can inhibit the growth of other bacteria, particularly those called “cheater” bacteria above exhibiting weakness in organophosphorus mineralization [95]. In the experiments conducted by Jin et al., the secretion of *Streptomyces* was found to inhibit the growth of 14 species of bacteria, predominantly those exhibiting weakness in organophosphorus mineralization [95]. The presence of *Streptomyces* significantly improved the nutrient uptake and transport efficiency of the AMF, optimizing the structure and function of the interstitial microbial community, and improved soil biological quality with AMF.

*Streptomyces* employs a dual mechanism of “metabolism-defense,” characterized by the up-regulation of *phoD* and *pstS* genes, engagement in C-P exchanges with AMF, and the secretion of antimicrobial substances, thereby providing a theoretical basis for constructing SynCom. However, the activity of SynCom decreased in low organic content soil. In the future, it is necessary to optimize the proportion of SynCom in co-cultivation by combining the characteristics of SynCom secretion of polysaccharides and the carbon source of AMF to maintain the stability of the community.

#### 3.2.2. Pseudomonas

Although there are many plant pathogens in the genus *Pseudomonas*, a strong synergistic effect of particular species of *Pseudomonas* on plants and AMF has been demonstrated (Table 3). It can have a significant impact on plant growth and especially disease control by synthesizing large amounts of antibiotics [101].

For example, *Pseudomonas fluorescens* collaborates with AMF, which significantly enhances *Pseudomonas fluorescens*’s ability to produce 2,4-diacetylphthalic acid (DAPG), a broad-spectrum antimicrobial that disrupts the cell membranes of various phytopathogenic bacteria, leading to their death. *Pseudomonas fluorescens* is regulated by AMF, and changes in the expression of related genes contribute to AMF colonization, soil mineralization, and antibiotic production. When working with AMF, *Pseudomonas fluorescens* has notably improved its plant growth-promoting functions compared to AMF alone, such as a significant increase in DAPG production when associated with *Ggt*, a root rot fungus of wheat, and a notable increase in plant height and water content when inoculated in combination with AMF [102,103].

*Pseudomonas putida* UW4 modulates plant ethylene via ACC deaminase and, along with AMF *Gigaspora rosea*, promotes plant growth, increases root ACC deaminase, and aids AMF colonization. Dual inoculation outperforms single inoculation in boosting biomass, root length, and leaf growth area [104]. Dual inoculation of *Pseudomonas aeruginosa* with AMF showed significant benefits under drought stress, improved the plant antioxidant defense system, increased drought tolerance, and synergistically boosted plant resilience to drought stress [105,106]. These studies imply that *Pseudomonas putida* and *Pseudomonas aeruginosa* collaborate with AMF via distinct mechanisms in modulating phytohormones and augmenting antioxidant capacity. Additionally, they complement the role of *Pseudomonas fluorescens* in producing antipathogenic compounds, thereby fostering plant growth and health.

**Table 3:** Major plant-promoting bacteria of the genus *Pseudomonas* and their main roles [104,107,108,109].

[Species Name	Main Effect for Plant-Promoting
*Pseudomonas flurescens*	• Secretion of antimicrobial substances (e.g., pseudomonasins) to inhibit pathogenic bacterian [107] • Synthesis of phytohormones (e.g., gibberellins) to promote root growth
*Pseudomonas putida*	• Antibacterial secretion of pseudomonasin • Synthesis of gibberellin-like hormones for plant uptake [104]
*Pseudomonas aerginosa*	• Synthesizes antibiotics (e.g., Pseudomonas aeruginosa) and antimicrobial polysaccharides • Secretes indoleacetic acid to promote growth and enhance plant resistance [110]
*Pseudomonas nitroreducens*	• Secretion of chitinase and nitrate pseudomonasin to break down pathogenic bacterial cell walls • Production of cytokinins to promote plant growth [108]
*Pseudomonas protegens*	• Produces antimicrobial secondary metabolites: Secretes compounds (e.g., Pyoluteorin, DAPG) inhibiting bacterial/fungal pathogens [111] • Promotes plant growth: Enhances shoot/root biomass, even under pathogen stress like nematode infection [112] • Modulates plant-associated microbiota: Alters rhizosphere/endosphere microbial communities, influencing pathogen suppression or facilitation [112]

#### 3.2.3. Rhizobia

*Rhizobia* constitute a group of bacteria that establish nitrogen-fixing nodules with specific host plants, including *Rhizobium*, *Sinorhizobium*, *Bradyrhizobium*, and *Azorhizobium.* Various species of *Rhizobia* may confer distinct and diverse probiotic effects, encompassing, but not limited to, efficient nitrogen fixation, phytohormone secretion, enhancement of plant resistance to biotic and abiotic stresses, and modification of adverse environments.

*Rhizobium* plays a vital role in nitrogen fixation, supplying essential nitrogen to plants while improving soil structure and increasing soil fertility. This enhances agroecosystem stability and sustainability. AMF-based SynCom will inevitably encounter *Rhizobium* in practical use. When AMF and *Rhizobia* coexist, their mutualistic relationship significantly enhances their plant-promoting functions. AMF demonstrates substantial mineralization and translocation of organic phosphorus in the soil, while *Rhizobium* provides plants with vital, accessible nitrogen, mainly through the fixation of airborne nitrogen [113]. The differences in nutrient types and sources create a mutually beneficial mechanism that greatly improves the efficiency and overall uptake of nutrients by the microbiome, encouraging plant growth. Qin et al. found that combining AMF and *Rhizobia* significantly increased soil ammonium and nitrate nitrogen. In the combined group, ammonium nitrogen rose by 20% compared to single inoculation, and effective phosphorus increased by 15% [114]. *Rhizobium*–AMF interactions also enhanced plant resistance. Wang et al. demonstrated that mixed inoculation increased antioxidant enzyme activities, reduced Cd-induced lipid peroxidation and ROS stress, thereby boosting plant resistance to Cd [115].

*Bradyrhizobium* facilitates plant growth and biological nitrogen fixation through a tripartite symbiosis with AMF on leguminous hosts. In greenhouse experiments involving cowpea (*Vigna unguiculata*), co-inoculation of a native *Bradyrhizobium* isolate (BspARI#1) and a commercial AMF mixture (comprising *Rhizophagus intraradices* and *Funneliformis mosseae*) resulted in a 45–65% increase in shoot biomass and a doubling of nodule numbers compared to either single inoculation or absence of inoculation [116]. Similarly, a parallel study with soybean (*Glycine max*) and *Bradyrhizobium japonicum* 532C showed that the symbiotic relationship developed faster when the fast-colonizing AMF *Glomus clarum* was present. By ten days post-emergence, 30% more nodules were observed than with *Gigaspora margarita* or bacteria alone [117]. During flowering, undisturbed soil with intact mycelium increased nitrogen fixation by 17% compared to disturbed soil, regardless of the AMF species involved. In summary, these findings show that interactions between *Bradyrhizobium* and AMF support legume growth by speeding up nodule formation and improving nitrogen uptake, with *G. clarum* offering better early colonization and functional complementarity to bacteria.

*Sinorhizobium meliloti* enhances plant development and mitigates salt stress, particularly when combined with *G. mosseae*. Zhu et al. (2016) conducted a factorial experiment on alfalfa cultivars with four salinity levels, comparing single and dual inoculations. Dual inoculation boosted nodulation, mycorrhizal colonization, shoot proline, and forage yield under saline and non-saline conditions, outperforming single inoculations. This synergy results from *S. meliloti*’s host-specific nodulation, improving nitrogen fixation, and *G. mosseae*’s enhancement of phosphorus uptake and osmolyte levels accumulation [118]. Collectively, they establish a symbiosis that enhances plant resilience by improving nutrient acquisition and stress-related metabolites such as proline, thereby supporting *S. meliloti*’s dual role in promoting growth and alleviating salt stress.

Moreover, in the spatial structure of nutrient uptake, the mycelial network of AMF overlaps and intertwines with the rhizobial network of *Rhizobium*, increasing the density of nutrient transport pathways in the soil [119]. AMF expands its mycelium beyond the inter-root zone under the stimulation and nutrient feeding of rhizobia, forming common mycelial networks (CMNs) to transfer nutrients among different plants and help them absorb nutrients such as phosphorus [113]. Meng et al.’s experiments were conducted in a root isolation mode, and the mixed inoculation of AMF and rhizobacteria in the no-barrier treatment improved by 93.7% relative to the solid barrier [120].

#### 3.2.4. *Bacillus* spp.

**Figure 5:**
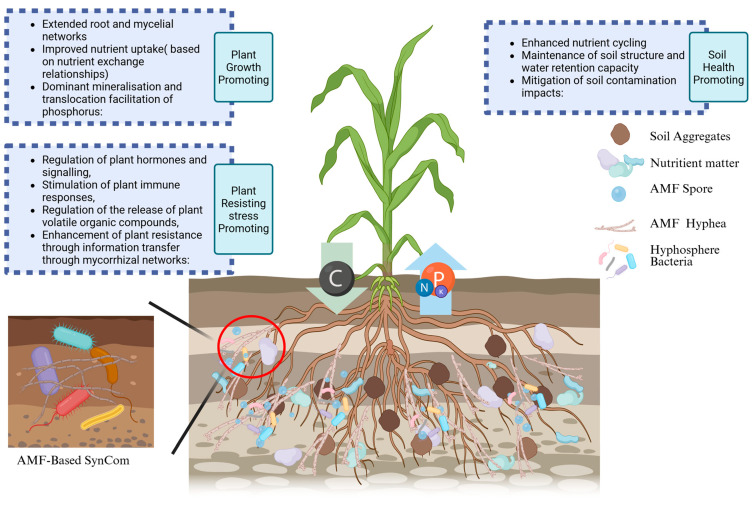
A Schematic diagram of the effect of AMF-based SynCom plant promotion.

*Bacillus* spp. is among the most extensively studied and applied bacteria for their ability to enhance plant growth and promote soil health (Figure 5). These bacteria are known for producing various bioactive compounds, such as lipopeptides, polyketides, and siderophores, which can directly inhibit the growth of plant pathogens and improve nutrient uptake by plants [121]. When combined with AMF, *Bacillus* spp. can significantly boost the efficiency of nutrient cycling and plant growth promotion.

The interaction between *Bacillus* spp. and AMF is multifaceted. *Bacillus* spp. can enhance AMF colonization by improving soil structure and nutrients. For example, *Bacillus subtilis* has been shown to produce surfactins, which can enhance the penetration of AMF hyphae into plant roots, thereby improving mycorrhizal association colonization [122]. Additionally, *Bacillus* spp. can indirectly support AMF by producing organic acids and other compounds that solubilize soil minerals, making phosphorus and other nutrients more available for uptake by both the plant and the AMF [123].

In SynComs, *Bacillus* spp. plays a vital role in improving the stability and function of the community. Their ability to produce bioactive compounds not only shields the community from pathogenic bacteria but also encourages the growth and activity of AMF. For example, co-inoculation of *Bacillus subtilis* with AMF has been shown to increase plant biomass and nutrient uptake more effectively than either treatment alone [85]. Moreover, *Bacillus* spp. can modulate the expression of genes related to nutrient uptake and stress response in both AMF and the host plant. This genetic regulation can further enhance the efficiency of nutrient cycling and improve the overall health of the plant-microbe system [122]. The integration of *Bacillus* spp. into AMF-based SynCom can thus provide a robust and multifunctional microbial consortium.

### 3.3. Synergistic Effects of AMF with Multiple Microbes and Their Combined Effects on Plants

Building upon prior analysis of synergistic interactions between recommended rhizosphere or hyphosphere strains within AMF-based SynCom, this study further examines the functional impacts of integrated SynCom assemblies—co-colonizing multiple AMFs and PGPRs—on plant growth and stress resilience. This synergistic effect of multi-species co-colonization has been validated in several studies, providing tangible evidence for the application of the AMF-based SynCom.

The considerable function of AMF-based SynCom in plant growth promotion was verified. In a study on olive tree (*Olea europaea*), Mechri et al. used AMF with a variety of PGPR (containing AMF: *Rhizophagus. irregularis* DAOM 197198; PGPR: *Streptomyces beta-vulgaris* strain B11: I-3639, *Bacillus megaterium* strain AGN01: I-4466, *Burkholderia. cedrus* strain AGN02: I-4360) and significantly improved olive tree growth [124]. The co-colonization of AMF with several PGPRs showed a significant improvement in various nutrient uptake metrics compared to the other groups; the various nutrient uptake indices showed significantly improved characteristics, including considerably increased levels of PLFA 16:1ω5 and NLFA 16:1ω5 in the inter-root and root systems of olive trees, as well as a significant increase in the total PLFA content of the soil in the inter-root zone of olive trees. Microbial communities at all levels were significantly altered, and significant alteration of nutrient element content in the root system of olive trees was also found. Nitrogen (N), phosphorus (P), iron (Fe), and manganese (Mn) were the most dominant macronutrients and micronutrients in the olive root system, and their contents were significantly increased by the inoculation treatments; the phenolic profile of the olive root system was also altered. All these phenomena suggest that the co-colonization of AMF and diverse PGPR not only enhances the colonization of AMF in the olive root system but also improves the availability of soil nutrients through the structure of the inter-root microbial community in this century.

The great potential of AMF colonization with various microorganisms in improving the adaptive capacity of plants to adversity was demonstrated. Hashem et al. used a variety of AMFs (including *Claroideoglomus etunicatum*, *Rhizophagus Intraradices*, and *Funneliformis mosseae*) along with *Bacillus subtili**s* inoculated under salt-stressed conditions into the *Acacia gerrardii* inter-root for co-colonization [125]. The findings indicated that the simultaneous colonization of various AMFs alongside *Bacillus subtilis* substantially enhanced the resistance of *Acacia gerrardii* under conditions of salinity stress. *Bacillus subtilis* facilitated AMF colonization within the roots by synthesizing cellulose and other pertinent substances. The synergistic effect between them also significantly reduced the negative effects of salt stress by decreasing the accumulation of harmful ions in the plant.

In summary, the construction of AMF-based SynCom is more than a description of the relationship between a single strain and AMF, and the significant synergistic effect of multi-species combinations better shows the great potential and broad prospect of AMF-based SynCom in sustainable agriculture and ecological restoration.

## 4. Limitations of AMF-Based SynCom Research and Application

### 4.1. Limitation in AMF and SynCom Field

At this preliminary stage, AMF-based SynCom exhibits several limitations and deficiencies. While it leverages the strengths of both AMF research and SynCom technologies, it also faces inherent challenges from both domains. Despite significant progress in AMF research, detailed molecular mechanisms, gene regulation, signaling pathways, and their interplay remain poorly understood [126]. Insufficient understanding of AMF makes it difficult to accurately design and build an efficient SynCom, which may always have unanticipated antagonistic effects, thus reducing the actual effectiveness and validity of SynCom. Additionally, while AMF-based SynCom can potentially expand environmental adaptability through controlled strain succession, specific roles and adaptability ranges need further field validation [127].

Advanced patterns of interactions are under-explored, i.e., the detailed dynamics of interaction networks formed by multiple microorganisms are not clearly understood. A quantifiable model for the metabolic reciprocity between AMF and its associated bacteria has yet to be established. For instance, the spatiotemporal dynamics of cross-kingdom signaling molecules such as lipopeptides remain understudied. This can lead to insufficient control over the functional prediction and precise construction of SynCom, putting SynCom bacteriophages at risk of being ineffective. Moreover, the lack of dynamic regulatory technologies that can adjust microbial community compositions in response to environmental changes is a significant obstacle. The failure of temperature-sensitive gene exemplifies this switches under field conditions. The absence of precise control over SynCom composition is partly due to the functional randomness that emerges when a designed SynCom is exposed to specific contexts. When a designed SynCom is introduced into a particular environment, its actual function may deviate from the initial expectations. This not only limits the environmental adaptability of SynCom but also poses challenges in achieving consistent functionality across different environments. Furthermore, current methodologies are insufficient for precisely regulating microbial community composition and function, which leads to inconsistencies in production scale, product functionality, and application convenience.

### 4.2. Perspectives

Given the above limitations, AMF-based SynCom still requires deeper and broader exploration and research to become a widely used tool for ensuring global food production and soil quality assurance in the face of drastic climate and food production changes in the coming years. Here, we envision some strategies to achieve this goal.

(1) Utilize gene editing technology to precisely modify microbial key genes, enabling them to perform functions similar to or even identical to those of core microorganisms, which are a group of microorganisms that play a crucial role in plant-associated microbial communities. For instance, the overexpression of *phnAc* (pyrene dioxygenase) in *Paracoccus aminovorans*, along with the elevated expression of *nifHDK* (nitrogenase complex) in *Azotobacter chroococcum*, facilitates synergistic pyrene degradation in nitrogen-deficient soils, achieving a 90% removal rate of pollutants through cross-feeding metabolic dependencies [128]. Similarly, the introduction of *mpd* (organophosphate hydrolase) into *Pseudomonas putida*, coupled with *pnpA*-expressing *Ochrobactrum* strains, enables complete pesticide mineralization within 28 days [129]. Additionally, the chromosomal integration of *czcA* (heavy metal efflux pump) in *Advenella* species enhances cadmium removal by 82% via rhizosphere cooperation [130]. By targeting key microbial traits, gene editing enables precise SynCom design and assembly. This approach directly overcomes limitations in controlling community composition and function, culminating in stabilized, functionally resilient SynCom [131,132].

(2) Omics science, with its integrative and high-resolution toolkit, offers unprecedented power to dissect formerly elusive dynamics within SynComs, such as stability maintenance and dynamic metabolic complementarity. Metagenomics, for example, enables the simultaneous tracking of community structure and functional gene pools; Mataigne et al. (2023) reconstructed genome-scale metabolic models (GEMs) for 193 Arabidopsis root isolates and demonstrated that SynComs assemble around an optimal phylogenetic distance, predicting that merely 7–15 core strains can approximate the metabolic potential of the complete consortium [133]. Similarly, metatranscriptomics captures real-time gene expression; Kumar et al. (2023) employed RNA-seq-guided metatranscriptomics to analyze rice phyllosphere and rhizosphere communities, revealing selective transcription of *nifH* genes exclusively in the rhizosphere, thereby identifying spatially restricted nitrogen-fixation hubs [134]. Metabolomics, in turn, provides direct validation of metabolite exchange networks and nutritional dependencies. Gao et al. (2025) utilized stable-isotope tracing LC-MS to construct an isotopologue similarity network centered on glutathione, thereby experimentally confirming a novel transsulfuration reaction in which γ-Glu-Ser-Gly is directly synthesized from glutathione. This reaction was validated across 293T cell lines and mouse tissues, emphasizing glutathione’s role as a sulfur donor within SynCom-associated redox circuits [135].

(3) The effectiveness of efficient computational tools, notably Random Forest and Neural-ODE classifiers, has been demonstrated for processing high-dimensional microbial data, achieving AUROC values exceeding 0.8 in predicting colonization outcomes for *Enterococcus faecium* and *Akkermansia muciniphila* across 297 stool-derived in vitro communities. These models were trained using species-level relative abundance matrices, comprising approximately 160 taxa, and demonstrated superior performance compared to simpler diversity-based classifiers. This suggests that machine learning techniques are capable of identifying key taxa, such as *Faecalibacterium prausnitzii* and *E. faecalis*, which exert a substantial influence on invasion success. At the functional level, PICRUSt2 and Tax4Fun, when applied to 16S rRNA profiles, accurately predicted KEGG pathways with over 80% coverage for genes involved in butyrate and bile acid metabolism. This facilitated the selection of features to differentiate between probiotic and pathogenic *Enterococcus* strains. Bayesian networks further augment these predictive capabilities; for example, a Dirichlet-multinomial regression model achieved a Pearson correlation coefficient of 0.74 in forecasting the abundance of *A. muciniphila* after invasion, based on initial community composition. Consequently, the integration of these machine learning pipelines enables the rapid design of SynComs with predefined metabolic outputs, thereby eliminating the need for exhaustive pairwise culturing [136,137,138].

(4) Transitioning from SynComs to synthetic assemblages can be achieved by integrating AMF into a bacterial-only SynCom, which has been shown to enhance both community abundance and stability [139]. Similarly, supplementing SynComs with host-derived metabolites—such as root exudates that sustain microbial interactions—creates a host-metabolite microenvironment, effectively miniaturizing the community structure. This approach aligns with the concept of a “synbiotic portfolio,” where core functional microbes (probiotics) and their supportive host metabolites (prebiotics) co-function to buffer against seasonal fluctuations and soil microbial variability [140,141]. As a result, synthesizing symbionts may be the next step in SynCom to obtain greater efficiency and effectiveness.

## 5. Conclusions and Perspectives

**Figure 6:**
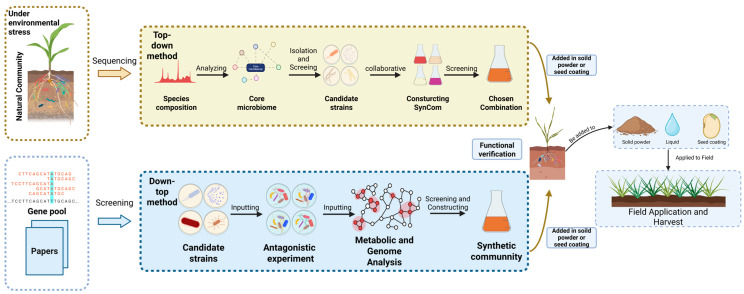
AMF-based SynCom Flow from Design to Field Experiments.

AMF-based SynCom represents a transformative approach to sustainable agriculture that harnesses the symbiotic capabilities of AMF to enhance plant growth, stress resistance, and soil health. Guided by both “top-down” and “bottom-up” methods and combined with the metabolic complementation approach, we can develop SynComs that sustain microorganism interactions through thorough strain screening, compatibility testing, and co-culture propagation. In its application, the AMF-based SynCom is usually used in solid form or as a seed coating on the plant’s inter-root soil or seed surface, aiming to ensure its colonization in the plant’s inter-root zone. The efficacy of the AMF-based SynCom can be evaluated post-deployment through the monitoring of microbial colonization and activity utilizing plant biomass assessments, soil mineral and biological indicators, and advanced methodologies such as Omics Science or PLEA mapping (Figure 6). By integrating AMF with functionally complementary microorganisms such as specific bacterial genera (*Streptomyces*, *Pseudomonas*, *Bacillus* spp.) or other AMF species, AMF-based SynCom exhibits superior stability, nutrient acquisition efficiency, and environmental adaptability compared to single-strain inoculants. The framework aims to advance AMF-based SynCom as vital tools for securing future food production amidst escalating climate and agricultural pressures.

## Figures and Tables

**Table 2:** Representative SynComs: Types, Construction Methods, Inoculation Approaches, and Colonization Outcomes.

Type of SynCom	Design and Construction Methods	Inoculation	Mechanisms and Effects
Suppressing maize seedling blight SynCom [35]	7 species selected from maize rhizosphere via selective media + 16S rRNA sequencing; assembled based on antagonism to *Fusarium*	Root drench at 10^7^ CFU/mL in sterile MS medium	The microbial community associated with maize roots was notably altered, with *Enterobacter cloacae* serving a pivotal function. The SynCom suppressed *Fusarium verticillioides* and reduced maize seedling blight incidence.
Suppressing tomato wilt SynCom [8]	93 bacteria + 74 fungi screened from tomato rhizosphere using metagenomics, NetShift + random forest modeling; cross-validated by 16S/ITS sequencing	Soil inoculation at 10^7^ CFU/g soil in ½ MS medium	The cross-border SynCom (CrossK) effectively suppressed tomato wilt disease (FWD), significantly reducing *Fusarium oxysporum f. sp. lycopersici* (FOL) populations. Various SynComs activated defense pathways in tomato plants, including JA and SA gene expression.
Mycorrhizal fungal-bacterial SynCom [61]	5 genera (*Pseudomonas*, *Flavisolibacter*, etc.) isolated from maize rhizosphere; co-inoculated with AMF	Soil inoculation at 10^8^ CFU/mL + AMF spores.	SynComs with mycorrhizal fungi significantly increased P and K concentrations in maize plants. Synergistic effect of SynCom with mycorrhizal fungi promoted maize growth as evidenced by higher dry weight and nutrient content.
Salt stress-tolerant SynCom [31]	15 strains from *Indigofera argentea* rhizosphere; selected for halotolerance via NaCl gradient plating	Seed coating in equimolar mixture	Pro inoculation led to lower Na^+^/K^+^ ratios, increased salt-resistant gene expression, and improved salt tolerance in tomato plants. The biomass of inoculated plants was notably greater than the control under salt stress, with less reduction in growth.

## Data Availability

No new data were created or analyzed in this study. Data sharing is not applicable to this article.
